# Interventional Effects of Weight-Loss Policy in a Healthy City among Participants with Metabolic Syndrome

**DOI:** 10.3390/ijerph16030323

**Published:** 2019-01-24

**Authors:** Hsu-Chih Tai, I-Shiang Tzeng, Yen-Ching Liang, Hsiu-Hui Liao, Chun-Hsien Su, Woon-Man Kung

**Affiliations:** 1 Department of Exercise and Health Promotion, College of Education, Chinese Culture University, Taipei 11114, Taiwan; soma8989@yahoo.com (H.-C.T.); tifa669@gmail.com (Y.-C.L.); yuhwa.su@gmail.com (C.-H.S.); 2 Department of Research, Taipei Tzu Chi Hospital, Buddhist Tzu Chi Medical Foundation, New Taipei City 23142, Taiwan; istzeng@gmail.com; 3 Health Section, Public Health Bureau of Miaoli County Government, Miaoli 35645, Taiwan; mlh24@tcmail.mohw.gov.tw; 4 Division of Neurosurgery, Department of Surgery, Taipei Tzu Chi Hospital, Buddhist Tzu Chi Medical Foundation, New Taipei City 23142, Taiwan; 5 Department of Surgery, School of Medicine, Buddhist Tzu Chi University, Hualien 97004, Taiwan

**Keywords:** metabolic syndrome, exercise, healthy eating, weight loss

## Abstract

This study aimed to establish a friendly environment of active living and healthy eating for citizens while promoting and increasing knowledge of healthy exercise. Acquisition of physical activity skills and citizens’ lifestyle changes result in reduction in rates of obesity and deaths related to underlying metabolism syndrome. This study used a non-experimental cross-sectional design to survey residents living in Taiwan’s rural Miaoli County. The inclusion criterion was positive screening for metabolic syndrome. In total, 2068 participants were recruited, and 1886 questionnaires (91.2%) were completed. An organization-spreading strategy and home convenient Lifestyles of Health and Sustainability tactic were applied to the assessment, promotion, evaluation, and planning of the project via an obesity-causing environmental scan, oriental synergy aerobics, acupuncture points massage, guide books, broadcasting, town-based “shape-it-station”, and a vending cart created to facilitate the acquisition of healthy foods. After the intervention, results revealed that health condition, regular exercise habits, diet behavior, metabolic syndrome cognition, and body weight became better than before. Appropriate promotion of healthy cities through public health measures may effectively reduce the threat of death due to metabolic syndrome, which in turn reduces overall, and represents successful control of a typical non-communicable disease.

## 1. Introduction

Metabolic syndrome (MetS) describes the aggregation of a spectrum of related medical conditions, including obesity, dyslipidemia, hypertension, and hyperglycemia [1], all of which are associated with an increased risk of cardiovascular disease (CVD) [2]. Despite the general principle underlying the concept of MetS to indicate an increased risk of CVD or diabetes, the definition of MetS remains unclear due to different defining criteria. In addition, definitions of MetS have been revised in the last few years, resulting in the current use of multiple definitions [3,4,5,6,7]. Diagnosis of MetS can be established if there is: (1) Abdominal obesity: a male’s waist circumference over 90 cm (35 inches), and a female waist circumference over 80 cm (31 inches); (2) High blood pressure: systolic blood pressure 130 mmHg or diastolic blood pressure 85 mmHg, or taking a medication prescription for hypertension; (3) Fasting high blood sugar: fasting blood glucose ≥ 100 mg/dL, or taking a medication prescription for diabetes; (4) Fasting high triglyceride: ≥150 mg/dL, or taking a medication prescription for hypertriglyceride; (5) Low-density lipoprotein cholesterol is low: male < 40 mg/dL, female < 50 mg/dL. MetS can be diagnosed if three of the above five components can be met.

In fact, the general concept is that a patient with MetS will experience some combination of conditions, such as obesity, dyslipidemia, hypertension, and insulin resistance or hyperglycemia. To successfully manage MetS, clinicians should identify related conditions. MetS is associated with an increased risk of CVD in the general population [8,9]. Furthermore, MetS is related to a higher incidence of type 2 diabetes [10,11]. MetS is also correlated with chronic kidney disease (CKD), thereby increasing attention from clinicians in selected populations [12,13,14]. CKD, a chronic illness similar to MetS, often progresses over a long period from mild reduction in glomerular filtration rate to more advanced pre-uremic states and eventually to the stage of irreversible kidney damage, requiring renal replacement therapy. From a clinical perspective, early CKD patients may experience symptoms such as fatigue, itching, nausea, anorexia, cramping, and muscle twitching. In its late course, asymptomatic or even symptomatic edema occurs. In each CKD phase, the relationship between MetS and CKD is usually treated as an isolated snapshot. 

The main purpose of this study was to use the MetS-CKD relationship as a point along each stage of the CKD spectrum to improve the diagnosis of MetS. This approach may help physicians to determine whether MetS is part of the etiology of CKD, the result of a common risk factor for MetS and CKD, or a completely unrelated entity.

According to various studies, knowledge and awareness of the warning signs of MetS may help people meeting MetS criteria to take early treatments [15]. Regarding to this scenario, Rogers (1975) proposed the Protection Motivation Theory (PMT), which is a widely accepted method and used as a general framework for predicting health behaviors and health-related interventions [16]. Generally, PMT is a theory of social cognition used to assess protection behavior and influence motivation. PMT investigates whether fear can enhance positive protection motivation through response costs, response efficacy, self-efficacy, perceived vulnerability, severity, and reward. 

Health education is the main basis for all preventive interventions concerning MetS among people. A policy-planning study has highlighted the role of age-friendly promotion in the modification of environmental factors or behavioral change [17]. With this background, the present study aimed to illustrate the modification of environmental factors or behavioral change via PMT-based educational intervention among citizens living in the rural Miaoli County in Taiwan.

## 2. Materials and Methods 

### 2.1. Participants

This quasi-experimental study was conducted in 2010 among MetS-positive residents of rural Miaoli County who were recruited through a community health assessment. From 2009–2010, public health nurses from various township health centers in the county’s 18 marginal cities and towns collaborated with volunteer residents to conduct screenings. Patients who received preventive health services or health checks (adult health check, elderly health check, integrated screening) at these clinics were screened for MetS and its high-risk groups. A list of participants with MetS and its high-risk groups were collected for the study population. Public health nurses contacted these participants, briefly explained the 12-week Lifestyles of Health and Sustainability (LOHAS) plan and asked their willingness to participate. A participant signature was required to indicate agreement with the plan.

Public health nurses worked with participants to implement the study questionnaire, which was developed by experts and famous scholars invited by the Miaoli County Sports and Healthy Eating Executive Committee. The questionnaire assessed demographics, exercise, diet, and weight loss. A total of 2068 participants completed this survey of MetS and its high-risk groups. The response rate was 91.2% (Table 1). The inclusion criterion of the study was MetS as defined by the Ministry of Health and Welfare (MOHW) of Taiwan. This study was conducted with the approval from the Institutional Review Board of Chinese Culture University (approval number 98018). Pre-test data were collected 1 week before the intervention, and post-test measures were collected 12 weeks after the end of the intervention.

We examined the inclusion criteria in 2 phases. Exclusion criteria include participant’s will of withdrawal or refusing to sign the informed consent. Phase I: collect MetS list from citizens. Our study collected a health checkup list of citizens through public health nurses from various township health centers. We conducted home visits to citizens who have received preventive health services or health checkups in 2008–2009. Next, we screened out citizens with MetS and completed the collection of a list with more than 2000 participants. Phase II: case contact included the following: (1) according to the list, we contacted the participants initially by phone, explained concisely the plan, and coordinated the time required for home visits; (2) public health nurses cooperated with volunteers according to previous appointments to explain the purpose and method of implementation. Then, the citizens with MetS are requested to participate in the 12-week LOHAS educational program (Table 2). The study subjects understood, agreed, and signed the informed consents. Finally, participants completed the survey questionnaires and fitness testing, and completed the data within one month during home visits.

### 2.2. Intervention Program

**Figure 1:**
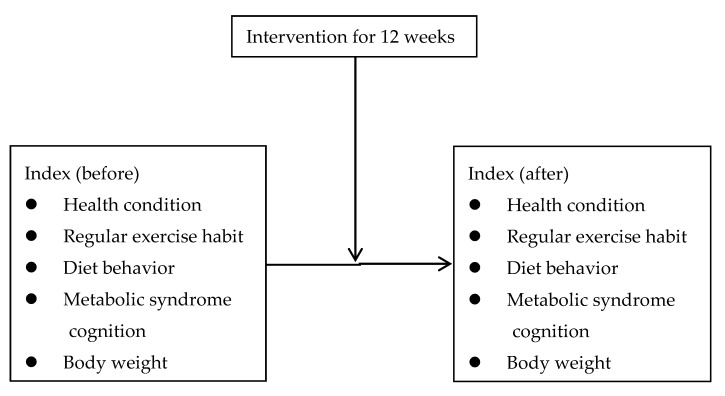
Study design.

The educational intervention was developed and implemented in 3 sessions within 12 weeks (Figure 1). Participants in the study group received the LOHAS educational program (Table 2). Each session was organized for small groups of 20–30 participants. Participants were educated by active learning methods, which included lectures, group discussions, and question–answer sessions after each educational session. The content of sessions focused on basic recognition of MetS, including epidemiology, signs and symptoms, risk factors, important early prevention, recommended screening methods, guidelines for blood smear screening, and the role of blood smear in early diagnosis. The educational content also included promotion of healthy eating and regular exercise. We defined the measurement of health condition standard score, exercise index score, diet behavior standard score, and MetS cognition for participants as follows:

(1) Health condition standard score = (Sum of the total score of the 3 specific questions)/15 × 100
(2) Exercise index score = Exercise intensity score × Exercise time score × Exercise frequency score
(3) Diet behavior standard score is divided into 2 parts:
  Outside food behavior score = (Sum of the total score of the 6 specific questions)/24 × 100
  Cooking at home behavior score = (Sum of the total score of the 8 specific questions)/32 × 100
(4) The standard score based on the recognition of MetS = (Number of correct answers for each question/Total number of selections for questions) × 100

### 2.3. Physical Status

Physical status was confirmed by the presence or absence of MetS. Subjects were classified according to the definition used in the questionnaire. Comorbidity history and age were collected via questionnaires and interviews.

### 2.4. Statistical Analysis

We explored the differences among different groups for characteristics using independent samples *t*-test and one-way analysis of variance (ANOVA) for continuous variables. A paired sample *t*-test is used for pre and post analysis, and McNemar’s test is applied for categorical variables on dependent data. We also performed post hoc analysis after one-way ANOVA analysis. Descriptive analyses used mean ± standard deviation (SD) for data presentation. The level of significance was set at *p* < 0.05. Statistical analysis was performed using SPSS version 24 (SPSS Inc., Chicago, IL, USA).

## 3. Results

The participants’ ages ranged from 20 to 85 years, with a mean age of 53.6 (±SD: 13.6) years in the participant group. A comparison of scores of healthy eating behaviors among subjects of different social demographic characteristics is shown in Table 3. Cross-tabulation analysis revealed that there were no significant differences in sex, marital status, religion, and working status between participant groups before implementation of the educational program (*p* > 0.05). However, there were significant differences in age, birthplace, educational level, and occupational class. We also performed the post hoc analysis presented in Table 3. 

Table 4 presents the comparison of four tracking terms regarding MetS screening behaviors before and after the educational program. According to the results, no statistically significant differences were found in the mean scores of health condition standard score in participant groups before versus after the intervention (*p* > 0.05). However, MetS cognition, sports index score, and healthy eating behavior standard score significantly increased after intervention in the participant group (*p* < 0.05). To assess the efficiency of the educational program in preventing MetS, cross-tabulation analysis was performed (Table 5). The results showed no significant differences between the two groups before the intervention (*p* > 0.05). According to the cross-tabulation, results of McNemar’s test verified that the null hypothesis is rejected (*p* < 0.05). Therefore, there is a statistically significant difference between past regular exercise and current regular exercise. The cross-tabulation showed that the situation is unchanged at 52.1% of the total (27.8% exercised regularly both in the past and currently; 24.3% neither exercised in the past nor currently; 21.6% exercised regularly in the past, but do not exercise regularly now; 26.3% did not exercise regularly in the past, but exercise regularly currently). The results showed that the educational intervention significantly increased the proportion of the population reporting regular exercising behaviors.

## 4. Discussion

This study describes knowledge, behaviors, and beliefs of residents with MetS in rural Miaoli County, which is located in northwestern Taiwan. While our results showed a relatively high level of knowledge and awareness regarding healthy eating and regular exercise, prevention of MetS was consistent with findings from other studies [18,19]. An important finding of the present study is that the mean of severity scores in the participant group showed a significant increase after the educational intervention. This improvement was recognized as a result of effective implementation of the educational program. Regarding improvement of regular exercising behaviors in the population, we attributed this to the short enrollment period affected by the effectiveness of the implementation of the LOHAS educational program (Table 2).

PMT assumes that severity and susceptibility of risk is assessed first, followed by evaluation of the efficacy of the recommended response by using a highly perceived threat measure [16]. In this theory, the threat assessment process is a combination of perceived severity and vulnerability minus perceived rewards. The educational intervention increased perceived threat by emphasizing the consequences of MetS and the benefits of screening behavior in the participant group. These results suggest that screening is more likely to be done if participants logically approach the benefits and understand the severity and harm of the disease (MetS) and its associated consequences. The mean scores of self-efficacy and response efficacy increased in our participant group after the intervention. For instance, do Rosário Pinto et al. report the effectiveness of educational intervention on the enhancement of self-efficacy and response efficacy of screening behaviors for MetS [20]. In addition, the mean scores of the participant group were significantly higher after the educational intervention. This result is consistent with the findings of other similar studies [20,21]. According to PMT assumptions, the response assessment process is the second assessment that people would consider when encountering a high-risk situation. Under such circumstances, people also assess their level of efficacy in addition to the recommended response. In the above theory, the response evaluation process is a combination of response efficacy and perceived self-efficacy minus response cost. However, barriers to screening behavior lead to reduced efficacy assessment. The identification of screening obstacles for MetS among participants in the participant group was one of the major objectives of the present study. 

Enhancement of females’ self-efficacy through presentation of successful examples, followed by the encouragement of those having regular screening behaviors were among other aims of the current study, leading to the improvements of efficacy in the participant group. Thus, the study staff were recommended to consider these points when executing the educational intervention. In the present study, there were no significant differences regarding the mean score of health condition standard after intervention in the participant groups, which is consistent with results in the literature [22]. However, our participant group was more likely to undergo MetS screening after educational intervention. Successful educational intervention increased the motivation (intention) of the participants. As observed in this study, when perceived threats and efficacy were at a high level, individuals appeared to be more motivated to control the risk and accept the recommended response. In other words, if people believe that the hazard is serious and the threat can be effectively blocked, they will be more cautious and knowledgeable.

In this study, our results suggested that health condition, regular exercise habit, diet behavior, MetS cognition, and body weight ameliorate after implementation of the intervention. Moreover, we found that the total deaths due to MetS in Miaoli County dropped by 0.39% after the intervention to 32.28% in 2011 from 32.67% in 2010. In addition to the all-causes mortality, CKD mortality showed a similar trend, decreasing from 2.87% in 2011 to 2.81% in 2012. These benefits are owed to the promotion of “Oriental Bio-Synergy” exercise, healthy eating, and healthy weight loss in this population.

### Limitations and Strengths

The limitations of the present study include the use of a self-reported method conducted according to the essence of quasi-experimental study design for data collection and statistical population that precluded the generalizability of the results. Therefore, similar studies are needed on other groups with MetS. Regardless of these limitations, this study has some strengths. This study was conducted in a marginalized population with high rates of MetS, which increased the likelihood of their eligibility to enroll and receive intervention. The advantages of using this strategy included acceptability, ability to deliver information, access to a marginalized population, modeling, and ongoing contact. However, this study only evaluates the intervention effect before and after analysis for the case group without blood sampling or blood pressure analyzing due to limited funding.

## 5. Conclusions

The weight-loss policy of healthy cities may indeed have a positive impact on the healthy living habits of MetS. The results showed that application of an educational intervention based on PMT would be an effective strategy for preventing MetS in residents of marginal rural areas. Therefore, application of this theory to health center education programs may lead to positive changes in the management of MetS prevention, which may have beneficial effects in the future. 

Our research project integrated public and civil departments, non-government organizations, and community resources to develop a model of “must move” and “healthy diet” in the strategy of sustainable development in Miaoli County, Taiwan. In conclusion, an appropriate health promotion project may effectively reduce the threat of death by MetS. This is an example of a successful project promoting the control of non-communicable diseases.

## Figures and Tables

**Table 1:** Response rate of 18 townships in Lifestyles of Health and Sustainability (LOHAS) program in Miaoli County, Taiwan.

Township		Pre-Test			Post-Test	
	*n*	%	Response Rate	*n*	%	Response Rate
Miaoli	296	14.3	100.0	236	12.5	79.7
Yuanli	160	7.7	100.0	126	6.7	78.8
Tongxiao	159	7.7	100.0	158	8.4	99.4
Zhunan	148	7.2	100.0	146	7.7	98.6
Toufen	243	11.8	100.0	177	9.4	72.8
Houlong	107	5.2	100.0	107	5.7	100.0
Zhuolan	48	2.3	100.0	48	2.5	100.0
Dahu	78	3.8	100.0	77	4.1	98.7
Gongguan	84	4.1	100.0	82	4.3	97.6
Tongluo	97	4.7	100.0	96	5.1	99.0
Nanzhuang	46	2.2	100.0	46	2.4	100.0
Touwu	114	5.5	100.0	114	6.1	100.0
Sanyi	104	5.0	100.0	89	4.7	85.6
Xihu	104	5.0	100.0	104	5.5	100.0
Zaoqiao	85	4.1	100.0	85	4.5	100.0
Sanwan	48	2.3	100.0	48	2.5	100.0
Shitan	80	3.9	100.0	79	4.2	98.8
Tai’an	67	3.2	100.0	67	3.6	100.0
Total	2068	100	100.0	1886	100	91.2

**Table 2:** Summary of educational sessions in the participant group in Miaoli County, Taiwan.

Sessions	Objectives	A Summary of Topics and Activities
Healthy eating	To increase awareness of the benefits of healthy eating	-Set one day per month as “vegetable and fruit healthy day”: media broadcasting promotion movie on the same day -Promote a healthy restaurant: eat local food and create new menu -Hold healthy eating contest: assist restaurant to prepare local food and develop healthy eating -Develop campus vegetable and fruit project: assist and design healthy eating project in college and university -Recruit vending carts selling fresh vegetable dishes to remote areas -Develop a convenient shopping environment for vegetables and fruit in offices -Provide free healthy menu: set out low calorie high fiber menu in high density population area -Develop organic vegetable and fruit planting: combine food scraps activity and assign planting section to provide free organic vegetables and fruit to citizens
Regular exercise	To increase exercise capacity gradually	-Provide healthy recreational sports map: set the flash board for the length and calorie expenditure of regular walking -Combination of exercise and breaking through the barricade: apply the exercise map resource (8 exercise spots set by 18 towns) and encourage students to participate the physical activity -Apply exercise map to civil departments; Miaoli County Government holds outdoor recreation and physical activity on a holiday
Healthy weight loss	To prevent the risk of metabolic syndrome	-Evaluation by questionnaire (cause of overweight environmental assessment, exercise and eat healthy investigation) and weight scale -Screening: (i) analyze and compare the improvements of participants’ recognition, attitude, and behavior after 12 weeks of public health education; (ii) screen participants’ body weight and follow-up each week; (iii) observe the effect of weight loss and the ratio of regain weight again. -Evaluate the status of improvements: (i) compare participants who exercise regularly before and after; (ii) compare participants who maintain a healthy diet before and after; (iii) analyze body weight before and after.

**Table 3:** Comparison of the scores of healthy eating behaviors among the subjects of different social demographic characteristics of the LOHAS program in Miaoli County, Taiwan.

Characteristics	Category	*n*	Mean	SD	t/F	Post Hoc
Sex	Male (1)	561	72.49	15.81	−0.02	
	Female (2)	1415	72.50	16.20		
Age	20–29 (1)	112	71.72	12.82	2.81*	(7) > (3), (4), (5)
	30–39 (2)	319	73.64	11.40		
	40–49 (3)	391	71.81	15.59		
	50–59 (4)	398	71.90	16.20		
	60–69 (5)	354	71.79	18.32		
	70–79 (6)	275	72.55	19.54		
	80–89 (7)	77	79.07	11.79		
Birthplace	Weinan (1)	691	71.73	16.46	2.67 *	(3) > (1), (2), (4)
	Hakka (2)	1137	72.89	15.96		
	Aboriginal (3)	62	77.03	11.92		
	Other provinces (4)	70	70.70	17.85		
Education level	Elementary school or under level (1)	564	73.01	17.44	2.51 *	(5) > (3)
	Junior high school (2)	274	73.52	15.29		
	High school (3)	337	70.74	17.05		
	College (4)	622	72.01	14.68		
	Graduate institute (5)	157	74.96	14.37		
Marital status	Celibate (1)	224	72.38	13.55	0.26	
	Married (2)	1560	72.36	16.40		
	Separated/Divorced (3)	41	71.21	14.62		
	Widowed (4)	144	73.40	16.48		
Religion	None (1)	563	73.75	14.09	2.38	
	Taoism (2)	730	72.42	16.07		
	Buddhism (3)	506	71.05	18.43		
	I-Kuan Tao (4)	36	73.55	12.93		
	Christian/Catholic (5)	102	74.60	11.92		
Working situation	None (housekeeper) (1)	710	73.72	16.00	1.69	
	Unemployed (2)	43	69.96	16.79		
	Retired (3)	219	71.37	20.03		
	Part time (4)	68	72.53	16.21		
	Full time (5)	891	72.14	14.54		
Occupational class	Public service (1)	190	70.17	16.72	4.52 ***	(2) > (4)
	Teach (2)	457	73.90	14.06		
	Agriculture (3)	77	74.28	13.86		
	Craft (4)	121	68.60	15.91		
	Business (5)	47	69.26	15.31		
	Freelance (6)	99	69.24	16.50		

* *p* < 0.05, *** *p* < 0.001.

**Table 4:** Comparison of scores before and after the LOHAS program in Miaoli County, Taiwan.

Index	*n*	Before	After	Paired T
Metabolic syndrome cognition	1612	56.80 ± 26.77	73.37 ± 23.95	23.12 ***
Exercise index score	1747	36.89 ± 26.44	41.01 ± 24.78	4.80 ***
Diet behavior standard score	1741	72.31 ± 16.24	75.64 ± 14.85	6.35 ***
Health condition standard score	1827	63.43 ± 12.75	64.17 ± 12.98	1.76

Different compliances in the 4 variables resulted in some participants lost to follow-up during the program. *** *p* < 0.001.

**Table 5:** Regular exercise measures in cross-tabulation.

Intervention	Pre-Test				Post-Test				Chi-Square
	No		Yes		No		Yes		
	*n*	%	*n*	%	*n*	%	*n*	%	
Regular exercise measures	710	50.6	694	49.4	645	45.9	759	54.1	6.09 *

Value of Chi-square obtained by comparing % in the variable values using McNemar’s test. * *p* < 0.05.
